# The use of deep learning technology in dance movement generation

**DOI:** 10.3389/fnbot.2022.911469

**Published:** 2022-08-05

**Authors:** Xin Liu, Young Chun Ko

**Affiliations:** ^1^School of Music and Dance, Huaihua University, Huaihua, China; ^2^Department of Education, Graduate School of Sehan University, Chonnam, South Korea; ^3^Department of Teaching Profession, Sehan University, Chonnam, South Korea

**Keywords:** deep learning, dance movements, action characteristics, sound characteristics, dance sequence

## Abstract

The dance generated by the traditional music action matching and statistical mapping models is less consistent with the music itself. Moreover, new dance movements cannot be generated. A dance movement generation algorithm based on deep learning is designed to extract the mapping between sound and motion features to solve these problems. First, the sound and motion features are extracted from music and dance videos, and then, the model is built. In addition, a generator module, a discriminator module, and a self-encoder module are added to make the dance movement smoother and consistent with the music. The Pix2PixHD model is used to transform the dance pose sequence into a real version of the dance. Finally, the experiment takes the dance video on the network as the training data and trained 5,000 times. About 80% of the dance data are used as the training set and 20% as the test set. The experimental results show that Train, Valid, and Test values based on the Generator+Discriminator+Autoencoder model are 15.36, 17.19, and 19.12, respectively. The similarity between the generated dance sequence and the real dance sequence is 0.063, which shows that the proposed model can generate a dance more in line with the music. Moreover, the generated dance posture is closer to the real dance posture. The discussion has certain reference value for intelligent dance teaching, game field, cross-modal generation, and exploring the relationship between audio-visual information.

## Introduction

Dance is a carrier of performing arts that widely communicates and spreads characteristic culture through human body movements. It is an effective means to reflect cultural diversity and national characteristics. For example, the dance performers' gestures, eyes, and facial expressions, which are ever-changing postures, can represent people's seven emotions and six sensory pleasures. It can even represent natural scenery such as heaven, earth, mountains, and rivers and natural phenomena such as day and night. It is closely connected with music in structure, artistic expression, and interpretation (Minturn and Fowlin, 2020). The continuous development of science and technology provides a broad development platform for deep learning (DL) technology. How to apply this technique to the generation of dance movements is one of the problems that educational circles pay attention to. The dance movements are different due to cultural and ethnic differences, and the follow-up DL technology will also study it. To make dance and music a better fit, choreographers need to create dance movements according to the characteristics of music. Choreographers listen to music, analyze music types, characteristics, inner feelings, or information, and then design corresponding dance movements according to music information. This whole process is called choreography. It is an art of collecting and organizing movement sequences based on music to reflect or express the dancer's thoughts and emotions (Moreu et al., 2020).

The traditional dance generation algorithm usually constructs a music action database containing massive music action pairs. When a music segment is used as input, it will be divided into several small music segments. Each music segment can find the most similar segment in the database. Then, the system can provide corresponding dance action candidates and combine them into a new dance action. In recent years, with the development and popularization of DL, the artificial neural network has been successfully applied to the generation of dance movements. The significant advantage of using DL for dance generation is that it can extract high-level features directly from the original data. In addition, deep neural networks can create new dance movements (Shang and Sun, 2020; Gao and Xu, 2021). Li (2020) proposed a deep neural network, which is trained from zero in an end-to-end manner and generates faces directly from the original speech waveform without any additional identity information. Their model is trained in a self-supervised way by using the audio and video features naturally aligned in the video (Li, 2020). Thomas and Blanc (2021) proposed a cross-modal generation model based on a cyclic generation countermeasure network by considering a cross-modal cyclic generation countermeasure network and combining different generated subnetworks into a network. It further enhanced the effect of mutual generation between music and images (Thomas and Blanc, 2021). Griffin (2021) tried to use an Encoder-Decoder neural network model to learn the corresponding relationship between the original audio and video. This model uses the joint embedding of face and audio to generate a synthetic speech face video frame. The input of the model is a still image and audio segment of the target face. Then, the lip video of the target face is synchronized with the audio output (Griffin, 2021). Elst et al. (2021) combined the convolutional neural network model and generation countermeasure network model to produce a real face sequence synchronized by two networks and input audio (Elst et al., 2021). The limitation of the above dance action generation method is that due to the use of the end-to-end model, the consecutive frame of the generated dance may not be smooth, making the visual effect of the generated dance worse. Moreover, the dance directly generated by the algorithm is often difficult to match the music.

The purpose is to enhance the consistency between the dance generated by the model and the music itself and to increase the smoothness and rationality of the long-time dance sequence. This exploration designs a dance generation algorithm based on DL to extract the mapping between sound and motion features. First, the prosody features and audio beat features extracted from music are regarded as music features. The coordinates of human skeleton key points extracted from dance videos are trained as motion features. Then, the basic mapping between music and dance is realized through the generator module of the model to generate a smooth dance posture. The discriminator module is used to realize the consistency of dance and music. The audio features are more representative through the autoencoder module. The improved Pix2PixHD model is used to transform dance pose sequences into a real-life dance. Finally, the loss function of the model and the generation results of cross-modal dance sequences are analyzed through experiments, which proves that the scheme of the dance automatic generation model based on DL is scientific and effective. The advantage of its future work lies in providing a method reference and rich theoretical basis for the generation of subsequent dance movements and expanding the fields involved in the current DL technology.

## Automatic generation algorithm of dance movement based on DL

### Related technology

OpenPose is one of the most popular multi-person pose estimation algorithms. Like many bottom-up methods, it first detects the key point coordinates of all people in the image, and then assigns the detected key points to each corresponding person. In practice, the OpenPose network first uses the first few network layers of Visual Geometry Group-19 (VGG-19) to extract features from images. Next, these features are transmitted to two parallel convolution layer branches (Simpson et al., 2014; Kim et al., 2021). The first branch is used to predict 18 confidence maps, each representing a joint in the human skeleton. The second branch predicts a set containing 38 Part Affinity Fields (PAF), describing the connection degree among joints. Next, a series of steps are used to optimize the predicted value of each branch. With the joint confidence graph, a bipartite graph can be formed between each joint pair. Then, the PAF value is used to delete the weak connections in the bipartite graph to detect the key points of the human posture of all people in the graph.

**Figure 1:**
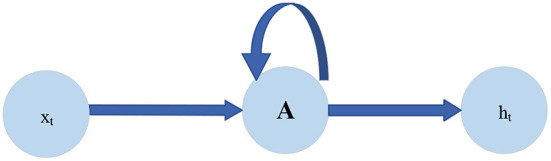
Structure diagram of RNN.

The traditional deep neural network cannot effectively solve the problem of time-series format data. The Recurrent Neural Network (RNN) solves this problem. RNN has a closed loop, which can continuously input the time-series information into the network layer at different times. This circular structure shows the close relationship between RNN and time-series data (Wang et al., 2021). Figure 1 is the structural diagram of RNN.

**Figure 2:**
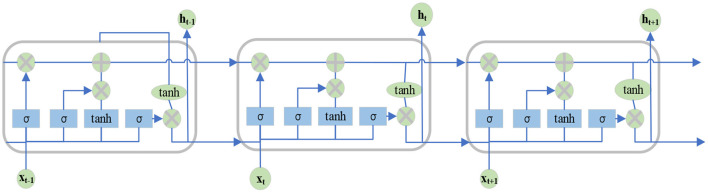
Schematic diagram of LSTM network structure.

Long-Short Term Memory (LSTM) neural network is a special type of RNN. It is designed to solve the problem of long-term dependence on RNN. The network modules of all RNN internal loops are the same. In ordinary RNN, the structure of the loop module is very simple. However, the special RNN of LSTM is designed to avoid long-term dependence, so different but similar structures are designed, in which each module has different structures. They interact in a quite special way. The key to LSTM lies in the state of cells in each network layer and the horizontal line passing through cells. The cellular structure is similar to a conveyor belt structure. Data run directly on the whole chain, with only a small amount of linear interaction (Noumi et al., 2021; Venskus et al., 2021; Yang and Lee, 2021). Figure 2 displays the details.

LSTM first decides what information to discard from the cell. This decision is controlled by the Sigmoid layer of the forget gate. For each element in the cell state *h*_*t*−1_, the forget gate outputs a number between 0 and 1 by inputting *h*_*t*−1_ and *x*_*t*_, which represents the percentage *f*_*t*_ of information retained from the previous cell state *c*_*t*−1_ to the current cell. 1 means “keeping all this information,” and 0 means “discarding all this information.” The updated equation of *f*_*t*_ reads:


(1) $$\begin{array}{l} f_{t}=\sigma \left(W_{f}\cdot \left[h_{t-1},x_{t}\right]+b_{f}\right) \end{array}$$

Then, the model will decide which new information to store in the cellular state. This step is divided into two parts. First, *h*_*t*−1_ and *x*_*t*_ are used to get *it* through an input gate to determine which information to update. Next, *h*_*t*−1_, *x*_*t*_, and a tanh layer are used to create a new cell vector candidate value ${\tilde{C}}_{t}$. This value may be updated to the cellular state. Equations (2) and (3) are *i*_*t*_ and ${\tilde{C}}_{t}$ updated equations.


(2) $$\begin{array}{l} i_{t}=\sigma \left(W_{i}\cdot \left[h_{t-1},x_{t}\right]+b_{i}\right) \end{array}$$

(3) $$\begin{array}{l} {\tilde{C}}_{t}=\tanh\left(W_{C}\cdot \left[h_{t-1},x_{t}\right]+b_{C}\right) \end{array}$$

Next, the old cellular state *C*_*t*−1_ is updated to the new status *C*_t_. The updated rule is to multiply the old state *C*_*t*−1_ of the previous time by the parameter *f*_*t*_ to forget part of the old cell state information. Then, the input gate added with a part of the candidate cell state ${\tilde{C}}_{t}$ information is used to update the state *C*_*t*_. C_ The updated equation of *C*_*t*_ reads:


(4) $$\begin{array}{l} C_{t}=f_{t}*C_{t-1}+i_{t}*{\tilde{C}}_{t} \end{array}$$

Finally, after updating the cell state, it is essential to determine the final output according to the input *h*_*t*−1_ and *x*_*t*_. The output will be based on the current cell state and some information will be filtered. First, the output gate of a Sigmoid layer is established to get the judgment conditions and determine which parts of the cell to output. Then, the cell state is passed through the tanh layer, so that the value of the output vector is between −1 and 1, and multiplied by the output gate. In this way, the final output result of the LSTM unit will be obtained. The updated equations read:


(5) $$\begin{array}{l} o_{t}=\sigma \left(W_{o}\cdot \left[h_{t-1},x_{t}\right]+b_{o}\right) \end{array}$$

(6) $$\begin{array}{l} h_{t}=o_{t}\cdot \tanh\left(C_{t}\right) \end{array}$$

**Figure 3:**
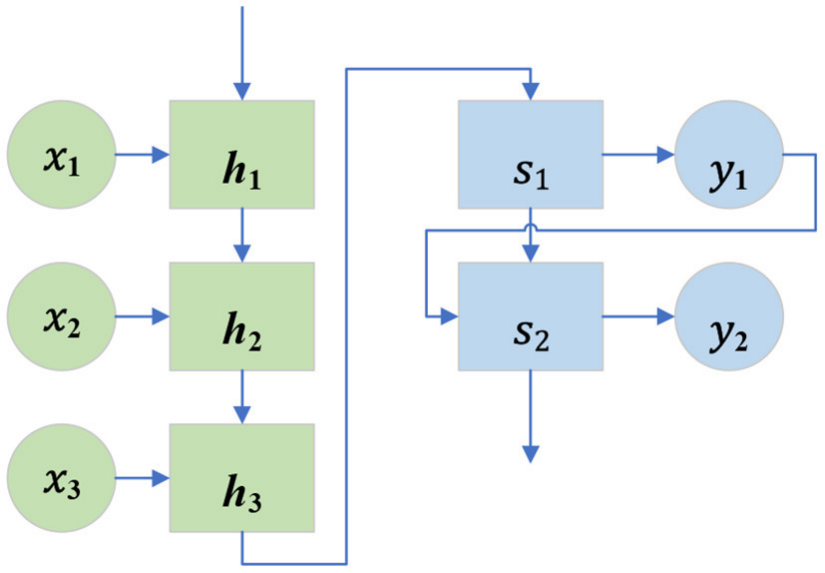
Network structure of the Sequence-to-Sequence model.

The attention model is first used in machine translation, and now it has become an important concept and tool in DL. The attention mechanism is an important part of the neural network structure, which has many applications in natural language processing, machine learning, computer vision, and other fields (Han et al., 2021). The Sequence-to-Sequence model is a kind of End-to-End algorithm framework. It is also a transformation model framework from sequence to sequence. It is applied in machine translation, automatic response, and other scenarios. It consists of encoder-decoder architecture. The encoder is an RNN that accepts the input sequence {*x*_1_, *x*_2_, … *x*_i_} (*i* is the length of the input sequence), and encodes it as a vector {h_1_, h_2_, … h_i_} of fixed length. The decoder is also an RNN, which takes a fixed-length vector *h*_*i*_ as the input to generate an output sequence {*y*_1_, *y*_2_, … *y*_j_}, where *j* is the length of the output sequence. At each time, *h*_*i*_ and *S*_j_ represent the hidden state of the encoder and decoder, respectively, which are called candidate state and query state, respectively (Luo et al., 2021; Zhou et al., 2021). Figure 3 is a schematic diagram of the network structure of the Sequence-to-Sequence model.

In the traditional encoder-decoder framework, the encoder must compress all input information into a fixed-length vector *h*_*i*_. Then, it is passed to the decoder. Using a fixed-length vector to compress the input sequence may lead to a large amount of information loss. Moreover, it cannot model the alignment between input and output sequences. The attention model can effectively solve these two problems. Its core idea is to introduce attention weight α into the input sequence to give priority to the location of relevant information to generate the output the next time. The attention module in the network structure with the attention model is responsible for automatically learning attention weight α_*ij*_, which can automatically capture the correlation between *h*_*i*_ and *S*_j_ (Gamal et al., 2020). These attention weights are then used to construct the content vector C, which is passed to the decoder as input. At each decoding position *j*, the content vector *c*_*j*_ is the weighted sum of all hidden states of the encoder and their corresponding attention weights. Equation (7) displays the details.


(7) $$\begin{array}{l} c_{j}=\overset{T}{\underset{i=1}{\sum }}\alpha_{ij}h_{i} \end{array}$$

Attention weight is learned by adding the feedforward neural network to the Sequence-to-Sequence architecture. The feedforward network learns a special attention weight α_*ij*_, uses *h*_*i*_ and *S*_j_ as the input of the neural network, and then learns the value of α_*ij*_ (Saka et al., 2021).

The autoencoder is a kind of neural network that uses a back propagation algorithm to iterate and make the output value equal to the input value. It first compresses the input information into the latent space representation and then reconstructs this representation into output. It is often used in dimensionality reduction and outlier detection (Samanta et al., 2020). Therefore, an autoencoder is actually a data compression algorithm, and its compression and decompression algorithms are realized through a neural network. It has three characteristics: (1) Data correlation. The autoencoder can only compress data similar to its previous training data. (2) Data loss. Compared with the original input, the output obtained by the autoencoder during decompression will have information loss. Hence, the autoencoder is a data lossy compression algorithm. (3) Automatic learning means that the autoencoder automatically learns from data samples, making it easy to train a specific encoder to input a specified class without any new work (Rahimzad et al., 2021; Verma et al., 2021).

Generative Adversarial Network (GAN) is a representative DL model. It makes the samples generated by the generated network obey the real data distribution through confrontation training (Yang et al., 2021). In GAN, there are two networks for confrontation training. One is the discriminative network. The goal is to judge whether a sample comes from real data or generated data from the network as accurately as possible and distinguish the generated data from the real data as much as possible. The other is the generative network. The goal is to generate real images as much as possible to deceive the discriminative network and make it unable to distinguish the samples from the source (Adamiak et al., 2021; Jeong et al., 2021). The final ideal result is that the model converges, and the discriminative network cannot judge the authenticity of the input samples, that is, the generated network can generate samples in line with the real data distribution.

Generally, image generation using GAN is to generate a random image according to random noise. Although the discriminator will judge the image's authenticity, the generated image is uncontrollable for the user. Pix2Pix model improves this problem. It uses paired data for training and realizes the mutual transformation of a group of images with some semantic relationship (Du et al., 2020). Pix2PixHD generates high-resolution and high-quality images based on Pix2Pix. With a source video and another target video, the goal is to generate a new video, so that the characters in the target video make the same actions as those in the source video (Boni et al., 2020). The task is divided into three stages to complete: the human posture detection stage, the global posture normalization stage, and the mapping from the standardized human posture to the target character. The model uses the open-source human pose detection framework to create the human pose skeleton map from the source video in the pose detection stage. In the global posture standardization stage, the model considers the difference in spatial position between the characters in the source video and the characters in the target video (Shin et al., 2020). Finally, the model designs a countermeasure generation network to learn the mapping from the standardized human posture skeleton map to the real person image of the target character.

### Model design

**Figure 4:**
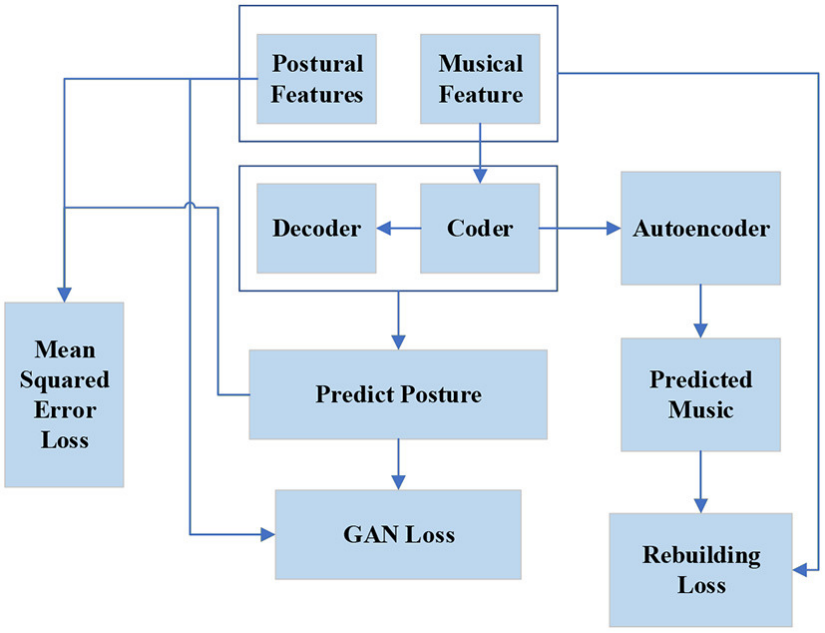
Schematic diagram of the network structure of the dance generation model.

The dance generation algorithm based on the DL model is committed to generating realistic dance movements and matching the music as much as possible. The timing characteristics of dance and audio data should be considered in the model design. The focus of the model design is the combination of dance and music and ensuring a good dance generation effect. First, according to music and dance data characteristics, a feature extraction scheme is designed, including the extraction of prosodic features and rhythm features. The dance generation model of generating dance posture according to audio features is designed based on the feature extraction scheme. The model includes the generator, discriminator, and autoencoder modules. Then, according to the dance pose sequence generated by the model, a scheme of real dance transformation is designed. Figure 4 shows the overall design of the model.

In Figure 4, first, the audio features and motion features are extracted from the dance data, then the audio features are input into the dance generator to obtain the predicted dance posture, and Mean Square Error (MSE) Loss is made with the real dance posture. The reconstructed audio features are obtained through the Autoencoder module and the Loss of audio reconstruction is made. The predicted and real dance posture are sent to the discriminator together, and the discrimination is conducted through the anti-loss training model.

**Figure 5:**
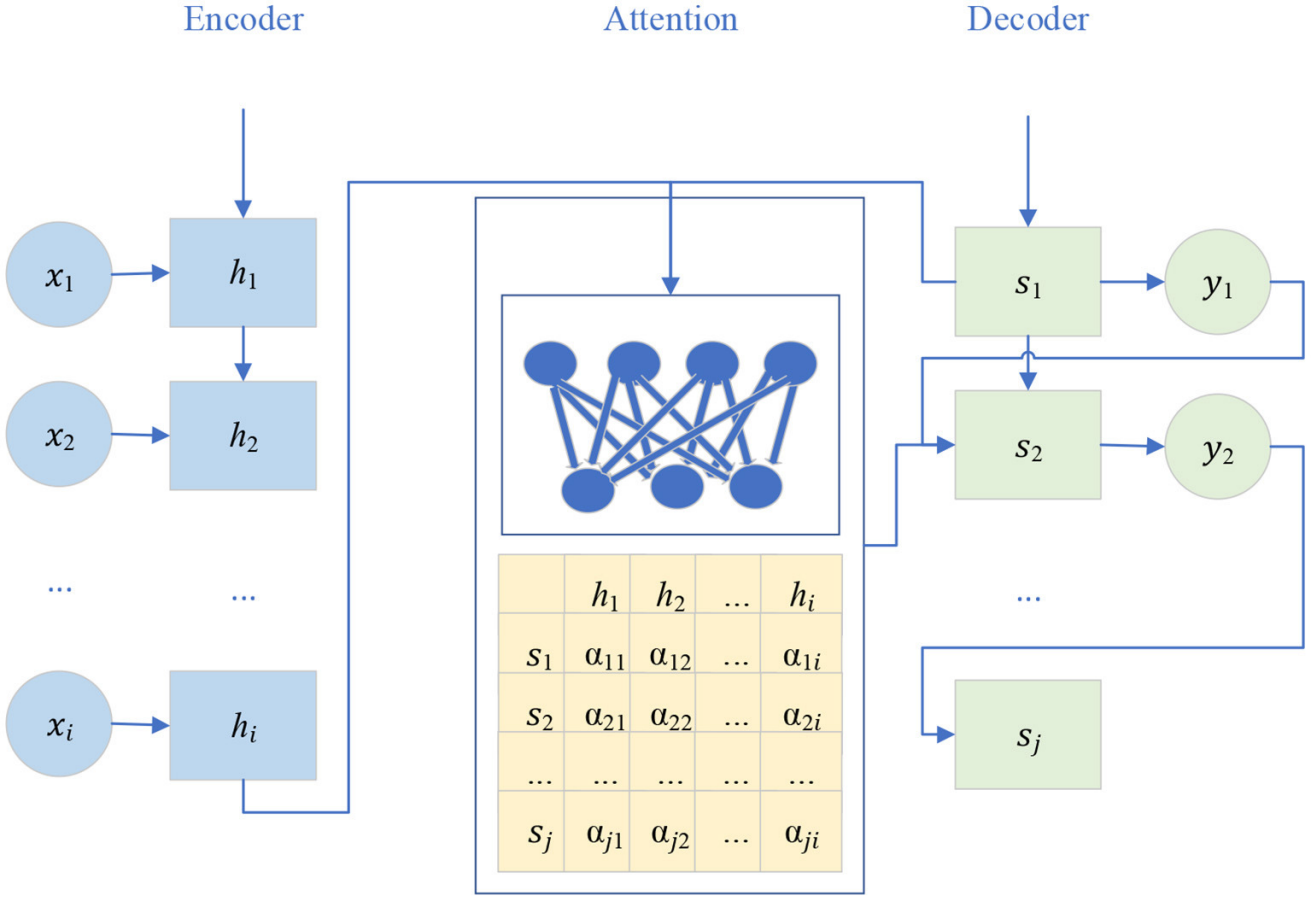
Schematic diagram of Sequence-to-Sequence model based on attention mechanism.

The Sequence-to-Sequence model has good sequence generation ability, so the generator module of the model is composed of a Sequence-to-Sequence model based on an attention mechanism. The generator model of the dance generation model with attention mechanism mainly includes three parts (Figure 5).

The encoder module is composed of multi-layer LSTM to extract long-term audio features. The input is the extracted audio feature vector and rhythm feature vector, and the output is the music context vector. The specific expression reads:


(8) $$\begin{array}{l} f_{C}=\text{ReLU}\left(F_{3}*\text{ReLU}\left(F_{2}*\text{ReLU}\left(F_{1}*E\left(X\right)\right)\right)\right) \end{array}$$

(9) $$\begin{array}{l} H=\;\text{EncoderRecurrency }\left(f_{c}\right) \end{array}$$

*F*_1_, *F*_2_, and *F*_3_ are three convolution kernels. ReLU is the non-linear activation on each convolution layer. *EncoderRecurrency* represents bidirectional LSTM. After feature extraction, the music feature sequence is first sent to three convolution layers to extract music context information, and then, sent to a bidirectional LSTM to generate the hidden state *H* of the encoder.

The encoder module and decoder module can calculate the hidden layer state *H* = {h_1_, h_2_, … h_i_} and hidden layer state *S* = {M_1_, M_2_, … M_j_}, respectively. h_i_ and M_j_ are the hidden states of the coding layer and the hidden states of the decoding layer at each time step. Then, the attention weight is calculated and assigned to the music context vector to obtain the audio feature vector after assigning the weight. Attention calculation occurs at each decoder time step. The custom score function is calculated for the target hidden state and each source state to generate attention weight. To reduce the potential sub-sequence repetition or omission in the decoding process, the cumulative attention weight of the previous decoding process is considered as an additional feature to keep the model consistent when moving forward along the input sequence. Therefore, the model uses the attention mechanism based on location sensitivity to expand the previous attention mechanism. Equation (10) displays the details:


(10) $$\begin{array}{l} e_{i,j}=score\left(s_{i},c\alpha_{i-1},h_{j}\right)=v_{a}^{T}\tanh\left(Ws_{i}+Vh_{j}+Uf_{i,j}+b\right) \end{array}$$

*f*_*i,j*_ is the position feature obtained by convolution of the previous attention weight α_*i*−1_. $v_{a}^{T}$, *W*, *V*, *U*, and *b* are the parameters to be trained. Through the attention calculation module, the attention weight between the hidden layer states h_i_ and s_j_ can be obtained.

The music training dataset is M = {M_1_, M_2_ … M_n_}. M_i_ is a sequence of audio feature vectors. The dance training dataset corresponding to music is *P* = {*P*_1_, *P*_2_ … *P*_n_}. *P*_i_ is the dance posture feature vector corresponding to M_i_. {M_i_,*P*_i_} is the training data of a sample pair. M and *P* are obtained from live dance videos through specific feature extraction schemes. The goal of the model is to train a dance generator *G* and realize the mapping relationship between M and *P*. Equation (11) displays the details:


(11) $$\begin{array}{l} L_{MSE}\left(G\right)=\frac{1}{N}\overset{N}{\underset{i=1}{\sum }}∥P_{i}-G\left(M_{i}\right){∥}^{2} \end{array}$$

Equation (11) shows the specific process of generator training. First, the model is trained on {M_i_,*P*_i_} and MSE Loss is calculated between the dance *G*(M_i_) generated by the model and the real dance *P*_i_. After the training, the corresponding dance posture sequence can be obtained for any given music input.

A human posture sequence is a time-series that changes constantly. Therefore, the difference between the front and back frames of human posture can reflect the change process of action, not just considering the fixed posture action in time sequence. Such a change process can better represent the characteristics of dance posture. The discriminator input is set as posture vector *P* = {*P*_1_, *P*_2_ … *P*_n_}, frame difference vector of the pose before and after is set as M={*P*_1_-*P*_2_, *P*_2_-*P*_3_, … *P*_n−1_-*P*_n_} and the audio feature vector is set as M = {M_1_, M_2_ … M_n_}. They are input into the discriminator together to judge whether the combination of pose vector and corresponding audio vector is true. Equation (12) shows the details:


(12) $$\begin{array}{l} \begin{array}{c} L_{GAN}\left(G,D\right)={𝔼}_{\left(P,M\right)}\left[\log D\left(\left(P,M\right)\right)\right]+\\ \frac{1}{2}{𝔼}_{M}\left[\log\left(1-D\left(\left(G\left(M\right),M\right)\right)\right)+\log\left(1-D\left(\left(W,M\right)\right)\right)\right] \end{array} \end{array}$$

Equation (12) reveals that the generator *G* receives the music feature vector M and generates the predicted dance posture through *C*, which is recorded as *G*(*M*). In the training phase, the generator *G* and the discriminator *D* are trained alternately in turn. *D* is a discriminative network, which is used to judge whether the generated dance is consistent with the music. ((*G*(*M*), *M*) sample pairs are set as a pair of negative samples. M is the other real dance pose that does not match the current audio. (*W, M*) is also set as a pair of negative samples. (*P*, M) is the sample pair composed of music and its corresponding real dance posture vector. It is set as a positive sample to train the discriminator. The output *D* (*G*(*M*), *M*) represents the probability of mutual fit between the predicted dance and music. The closer the value of *D* (*G*(*M*), *M*) is to 1, the better the fit between music and dance. The closer the output value is to 0, the more discordant the generated dance is with the music.

An Autoencoder module is added to the model to combine the music and dance posture more closely. The audio autoencoder module is a network designed for audio reconstruction. In the encoder stage of the generator module, the input audio features are encoded. When the coding is completed, in addition to the prediction of dance posture, the audio features are input into an autoencoder module and regressed with the audio features before coding. In this way, because the model's loss function needs to consider the regression loss of dance posture and the regression loss of audio reconstruction simultaneously, it can ensure that the dance posture will be more consistent with the music when the model is predicted. Moreover, the encoded audio feature vector can better represent the original audio with the model's training. The details are as follows:


(13) $$\begin{array}{l} f_{i}=Encoder\left(\mathrm{Concat}\left(M_{i},B_{i}\right)\right) \end{array}$$

(14) $$\begin{array}{l} M_{i}^{\sim }=Decoder\left(f_{i}\right) \end{array}$$

The audio autoencoder takes the audio feature vector M*i* and the beat feature vector M*i* as inputs. The LSTM network structure is used to encode the audio features. Besides, the same and symmetrical network structure is used for decoding. The basic model structure is shown in the equation. *M*_*i*_ is the original audio feature vector, *B*_*i*_ is the rhythm eigenvector, and $M_{i}^{\sim }$ is the reconstructed audio feature vector. *f* is a dimension-reduced audio representation extracted from the audio feature vector. *Concat* is a vector splicing operation. *Encoder* and *Decoder* are neural networks to be learned. The loss function of the audio autoencoder is defined as the Euclidean distance between the original audio feature *M*_*i*_ and predicted audio features $M_{i}^{\sim }$. The Equation reads:


(15) $$\begin{array}{l} L_{Recon\;}\left(\;Encoder,\;Decoder\;\right)=\frac{1}{N}\overset{N}{\underset{i=1}{\sum }}∥M_{i}^{\sim }-M_{i}{∥}^{2} \end{array}$$

To sum up, equation (16) displays the optimization objective of the model:


(16) $$\begin{array}{l} \underset{G}{\min}\underset{D}{\max}L_{GAN}\left(G,D\right)+\lambda_{1}L_{MSE}\left(G\right)+\lambda_{2}L_{Recon}\\ \left(Encoder,\;Decoder\right) \end{array}$$

λ_1_ and λ_2_ are training parameters.

### Experimental design

The dance synthesis results under different models and parameter settings are evaluated to verify the feasibility and effectiveness of the dance generation model. This experiment takes the dance data video screened and downloaded from the network as the experimental dataset for experiment and result analysis because the dance types of the platform are diverse and the research results will be more accurate. In addition, it is more convenient and quicker to choose. In the process of downloading, dance videos will also be screened to ensure that each type of dance is involved, so that their cultural characteristics can be captured from the dance. In the generator training phase, the model training times are set to 5,000 times. The model input dimension is 35, the encoder convolution layers are 3, and the maximum length of each convolution core is 5. The decoder dimension is 1,024, the prenet dimension is 256, the learning rate is set to 0.001, the gradient clipping threshold is set to 1, the weight attenuation is set to 1e-6, the batch size is set to 40, the seqlen is set to 125, and the optimizer is Adam. In the discriminator training phase, it is set that the discriminator is trained once every three training rounds of the generator. The learning rate of the discriminator is 0.001, the weight attenuation is 1e-6, and the optimizer uses Adam. The training set uses 80% of the dance data, and the test set uses 20% of the dance data. The experimental results are analyzed based on the model loss function and cross-modal dance sequence generation results. Table 1 shows the specific dance types selected.

**Table 1:** Selected dance types, nationalities, quantities and characteristics.

**Type**	**Detailed**	**Quantity**	**Style characteristics**
Mongolian dance	Drum dance	30	Bold and generous
	Chopsticks dance	30	
Hui dance	Banquet song	25	Cheerful and flexible
	Dancing flowers	25	
Uygur dance	Sanam	35	Warm, bold and delicate
	Dorang dance	35	
Yi dance	Cigarette box Dance	31	Positioning with “fire”
	Music and dance	31	
Zhuang dance	Shigong dance	29	Some labor actions
	Pole dance	29	

**Figure 6:**
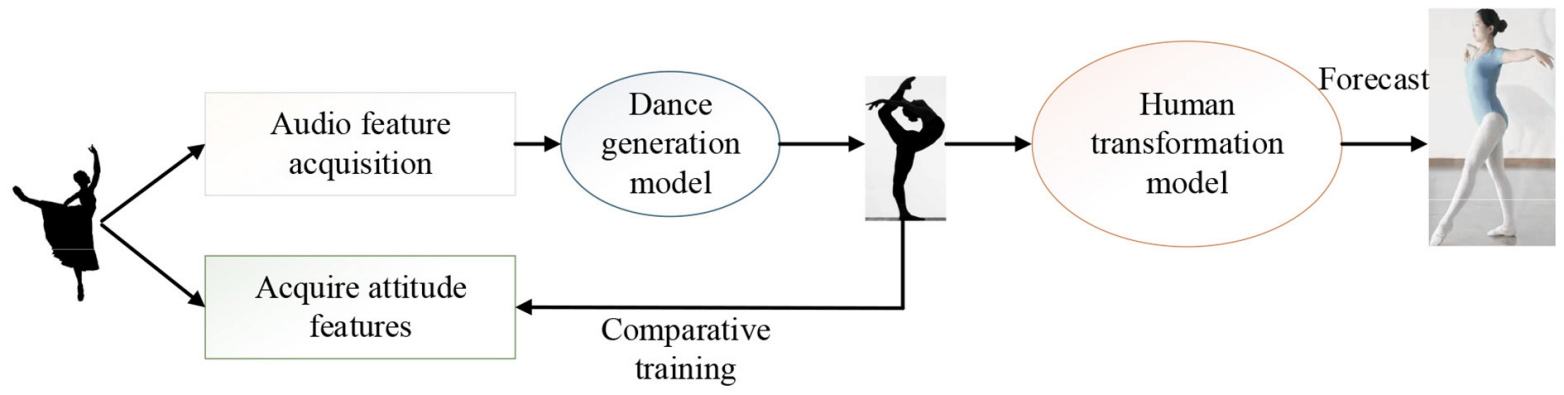
Experimental process of dance movement generation.

Figure 6 shows the specific experimental process.

## Experimental results and analysis

### Analysis results of the model loss function

**Figure 7:**
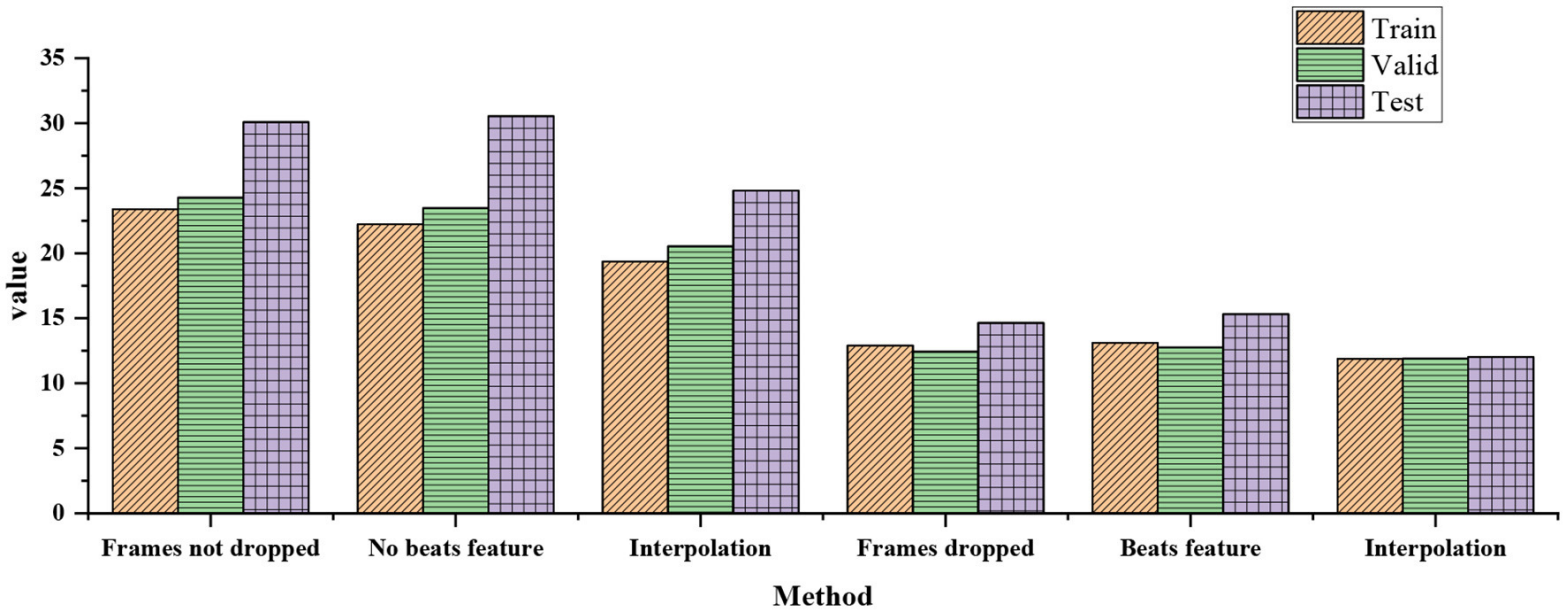
Influence of different data processing methods on final loss function in audio feature extraction stage.

In the stage of audio feature extraction, the influence of different data processing methods on the final loss function is analyzed, and the specific results are shown in Figure 7.

Figure 7 indicates that without filtering out the wrong data, the loss value of using the rhythm feature will be greater than that of not using the rhythm feature. This is also reasonable, because with the increase of the dimension of audio data, the noise may also increase, but the rhythm characteristics may be useful for the final generation effect of dance. The effect of using an interpolation function to supplement the missing values of dance data is better than not using it. It is very important to filter out the wrong data and it will enhance the final result.

**Figure 8:**
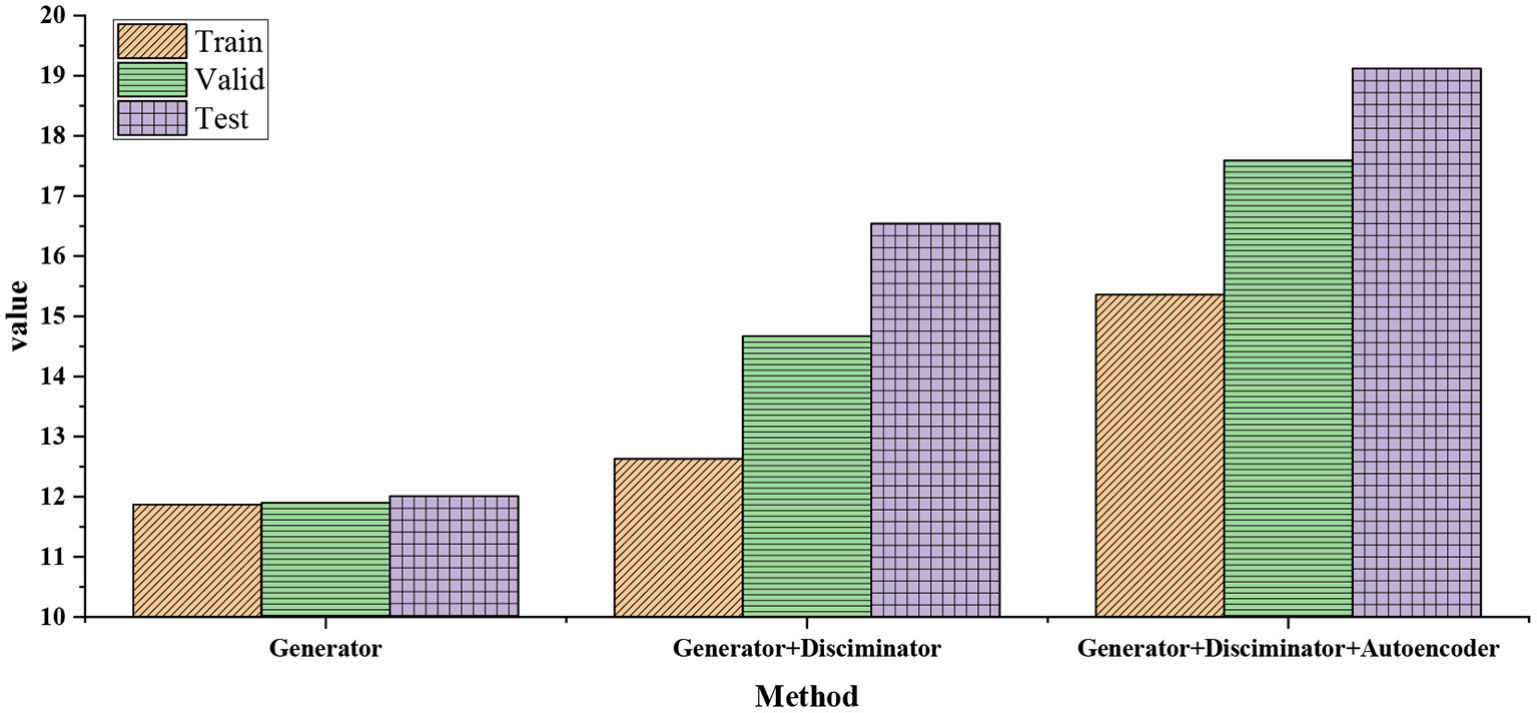
Impact of different modules on model loss in model building stage.

In the model-building stage, on the premise of filtering out the wrong data, using rhythm characteristics and interpolating the missing values, the impact of different modules on the model loss is analyzed. The results are shown in Figure 8.

As Figure 8 presents, the loss value with disciplinarian is slightly greater than that without disciplinarian. This can be expected because the confrontation training will make the generator generate a new sequence that does not necessarily match the original dance posture sequence, which may deviate from the real value of the dance posture predicted by optimizing Euclidean loss only. After the comparative analysis of the above three types of models, it can be seen that the autoencoder module has a significant impact on the loss of the model.

### Analysis of generation results of cross-modal dance sequences

**Figure 9:**
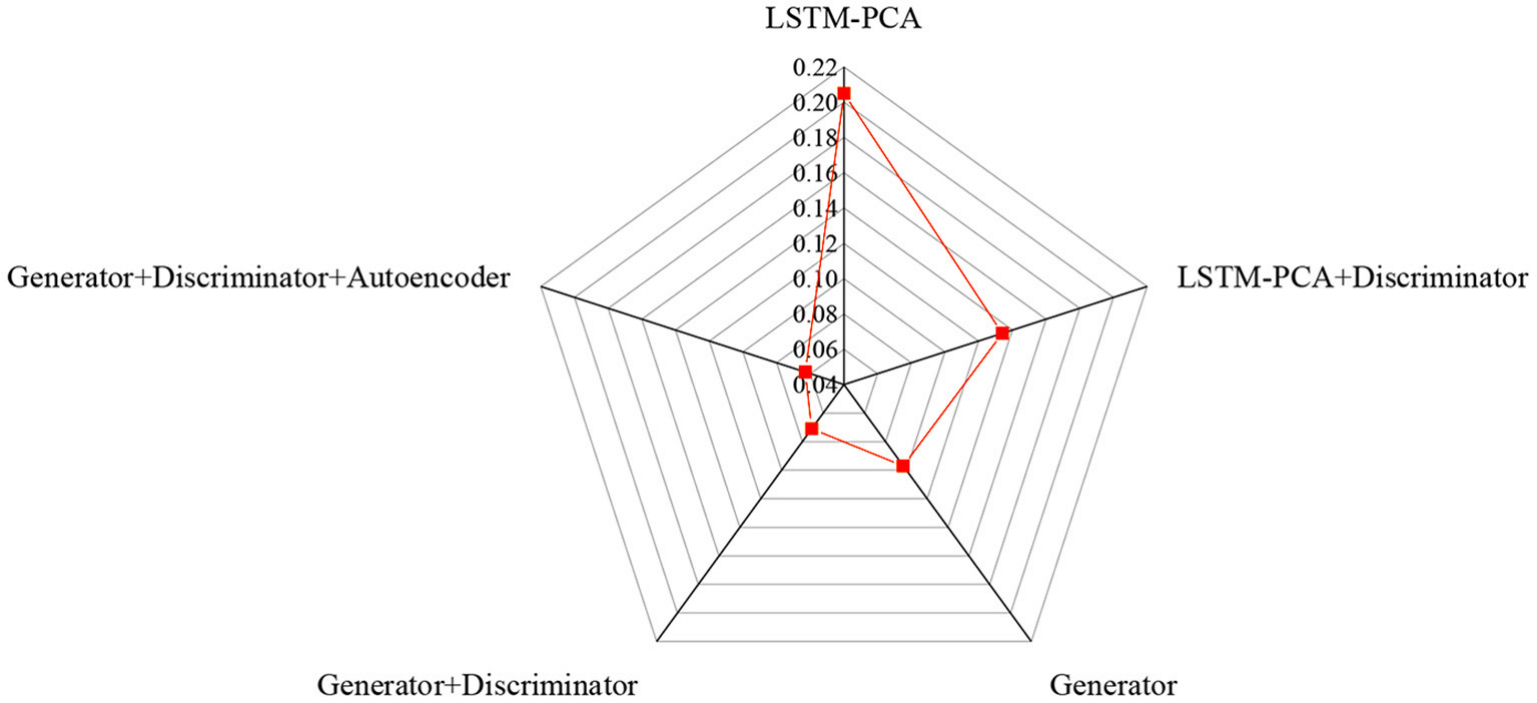
Similarity between robot-generated dance and real human dance.

First, the training set of the dance dataset is preprocessed. According to the settings of seqlen = 125 and batchsize = 40, music and dance sequences are segmented. Then, the segmented audio sequence features are extracted and projected into a dictionary together with their corresponding dance sequence. The dance generation model is used to generate dance from the music after segmentation. If the music segment does not appear in the previous training data, the most similar music segment is found in the audio feature vector dictionary obtained by the K-means algorithm and K-nearest neighbor algorithm. For K-means clustering, the number of clusters is set as k = 5. The similarity measure of the audio feature vector is Euclidean Distance. The similarity between the corresponding dance sequence and the generated dance sequence is calculated. If the music segment appears in the previous training data, the similarity between the music-generated dance sequence and the real dance sequence is directly calculated to measure the actual generation effect of the dance. Figure 9 shows the similarity between robot-generated dance and real human dance.

In Figure 9, the similarity between the robot-generated dance and the real human dance shows that the Generator+Disciminator+Autoencoder model has the best effect on the actual human dance generation. The dance effect of the network with a discriminator is better than that of the network without a discriminator. Moreover, the generator is superior to the generator of the LSTM series. To sum up, the dance generation model scheme based on Generator+Discriminator+Autoencoder can effectively extract music features and generate dance pose sequences that fit the music, which is feasible and effective.

**Figure 10:**
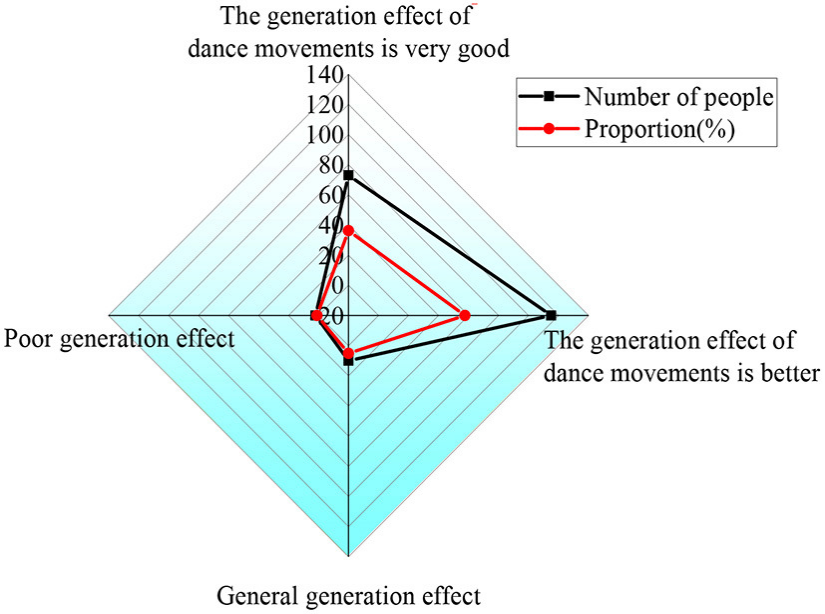
The dancer's evaluation of the dance action generation effect.

To verify the actual effect of the design model, 200 dancers are recruited to evaluate the effect of dance movement generation. Figure 10 shows the specific results.

Figure 10 shows that 36.26% of the dancers think the model has a very good dance movement generation effect; 57.61% of the dancers think that the model has a better generation effect; 5.13% of the dancers hold that the model has a general generation effect; and 1% of the dancers think that the generation effect is poor. After that, 1% of the dancers are paid a return visit. They believe that the reason why they think the generation effect is poor is that the video generated by the model will get stuck due to the impact of the network environment. However, most of the dancers recognize the designed model. Therefore, in the follow-up study, it is necessary to strengthen the improvement of the network environment.

## Discussion

With the development of science and technology, a variety of advanced technologies has been applied to the generation of dance movements. From the perspective of DL, this work studies the influence of this technology on the generation of dance movements. First, starting with the real dance scene, this work discusses and designs a DL-based dance action generation algorithm, which can extract the background music and dancers' actions. Second, it establishes the corresponding model. The selected data are applied to the model for research and analysis. Fink et al. (2021) tried to use the delayed LSTM artificial neural network to generate key points synchronized to audio, and then, another network was used to generate video frames based on key points. It is a network architecture that takes any text as input, and then, generates the corresponding voice and synchronous photo display pure synchronous video. Different from other published methods, their methods are only composed of fully trainable neural networks and do not rely on any traditional computer graphics methods (Fink et al., 2021). Wang and Tong (2022) further proposed a time consistency method for dynamic pixel loss. Compared with the direct audio-to-image method, this cascading method avoids fitting the false correlation between audio-visual signals independent of speech content. To avoid these pixel jitter problems, they also emphasized the network's attention to audio-visual related areas, and proposed a new attention mechanism with dynamically adjustable pixel-level loss. In addition, to generate clearer images with well synchronized facial motion, they proposed a new regression-based discriminator structure, which considers sequence-level and frame-level information (Wang and Tong, 2022). The above two scholars discussed their methods of generating dance movements from different angles. This study draws lessons from the effective methods and designs a model based on Generator+Discriminator+Autoencoder to generate dance movements. This study has a certain reference value for intelligent dance teaching, cross-modal generation, and exploring the relationship between audio-visual information.

## Conclusion

In the field of music-driven computer dance motion generation, there are many problems in the traditional music motion matching model and statistical mapping model. From the perspective of DL technology, this study studies the generation of dance movements, and mainly draws the following conclusions. (1) Taking 80% of the dance data as the training set and 20% of the dance data as the test set, it is found that in terms of the loss function, although the loss value based on Generator+Discriminator+Autoencoder model is higher than that of pure generator model, it can generate dance more in line with music. (2) In the evaluation of dance posture sequence, compared with other models, the dance posture generated by the model in this work is the closest to the real dance posture, whether for the music in the training set or the music in the test set. Experiments show that the scheme of the DL-based dance automatic generation model is scientific and effective. However, the amount of data selected here is less and there are some errors in the relevant test of the data. In addition, this study only analyzes the research of DL technology in dance movement generation but does not discuss the application of other technologies. In the future, a larger dance data set will be established to expand the training data, thereby, training a more representative dance movement generation model.

## Data availability statement

The raw data supporting the conclusions of this article will be made available by the authors, without undue reservation.

## Author contributions

Both authors listed have made a substantial, direct, and intellectual contribution to the work and approved it for publication.

## Funding

This work was supported by the Hunan Province Philosophy and Social Science Fund Project Research on the Art of Dance in Shangheyang Opera in Huaihua and its Creation and Communication (No. 19YBA269).

## Conflict of interest

The authors declare that the research was conducted in the absence of any commercial or financial relationships that could be construed as a potential conflict of interest.

## Publisher's note

All claims expressed in this article are solely those of the authors and do not necessarily represent those of their affiliated organizations, or those of the publisher, the editors and the reviewers. Any product that may be evaluated in this article, or claim that may be made by its manufacturer, is not guaranteed or endorsed by the publisher.
